# Nutritional status in perianal Crohn’s disease: are we underestimating the impact?

**DOI:** 10.3389/fnut.2023.1271825

**Published:** 2023-09-07

**Authors:** Bruno Augusto Alves Martins, Oswaldo de Moraes Filho, Ana Carolina Benvindo Lopes, Ricardo Jacarandá de Faria, Clíslian Silva, Gabriela de Oliveira Lemos, Dannilo Brito Silveira, João Batista de Sousa

**Affiliations:** ^1^Medical Sciences Postgraduate Program, School of Medicine, University of Brasilia, Brasília, Brazil; ^2^Department of Colorectal Surgery, Hospital Universitário de Brasília, Brasília, Brazil; ^3^IBD Unit, Department of Gastroenterology, Hospital Universitário de Brasília, Brasília, Brazil; ^4^Department of Nutrition and Dietetics, Hospital Universitário de Brasília, Brasília, Brazil; ^5^Multiprofessional Nutritional Therapy Team, Hospital Universitário de Brasília, Brasília, Brazil; ^6^Department of Colorectal Surgery, Hospital de Base-IGESDF, Brasília, Brazil

**Keywords:** (MeSH terms): inflammatory bowel diseases, Crohn’s disease, rectal fistula, nutritional status, malnutrition

## Abstract

Symptomatic perianal disease is common in patients with Crohn’s disease (CD), and perianal fistulas represent the primary form of anal involvement. This type of involvement is associated with a poor prognosis and a disabling course. The treatment is challenging and involves both surgical and medical approaches. Despite combined therapy, a significant portion of patients may still require proctectomy to control the symptoms. Consequently, investigating factors that may influence the outcome of perianal disease remains a priority area of research in CD. Nutritional deficiencies are well documented among CD patients with luminal forms of involvement and are closely related to poor clinical outcomes, therapy response, and postoperative complications. As a result, leading guidelines recommend regular nutritional assessment and correction of nutritional deficiencies in patients requiring a surgical approach. Despite these recommendations and the high rate of surgeries among CD patients with perianal disease, there is a shortage of studies addressing the real impact of nutritional status on the course and outcomes of perianal disease. This knowledge gap underscores the importance of further research to understand better and improve the management of perianal CD. This narrative review aims to provide an overview of nutritional status assessment and the influence of nutritional status on the outcomes of patients with perianal CD.

## 1. Introduction

Crohn’s disease (CD) is a chronic inflammatory disorder of the gastrointestinal tract, predominantly affecting the terminal ileum and colon. It is characterized by a relapsing and remitting course and can lead to progressive bowel damage (1). Symptomatic perianal disease is common in CD patients, with a prevalence of up to 50% over their lifetime (2, 3). Approximately 5% of individuals with CD experience anal disease as their initial manifestation, occurring even before luminal bowel inflammation (2, 4). Perianal fistulas are the primary form of anal involvement, with reported incidence rates of approximately 30%, followed by abscesses, fissures/ulcers, skin tags, and strictures (3). These lesions significantly impact the quality of life and are associated with fatigue and impairment of daily activities (5). In addition, the perianal disease is predictive of severe and disabling disease course (6, 7).

**Figure 1:**
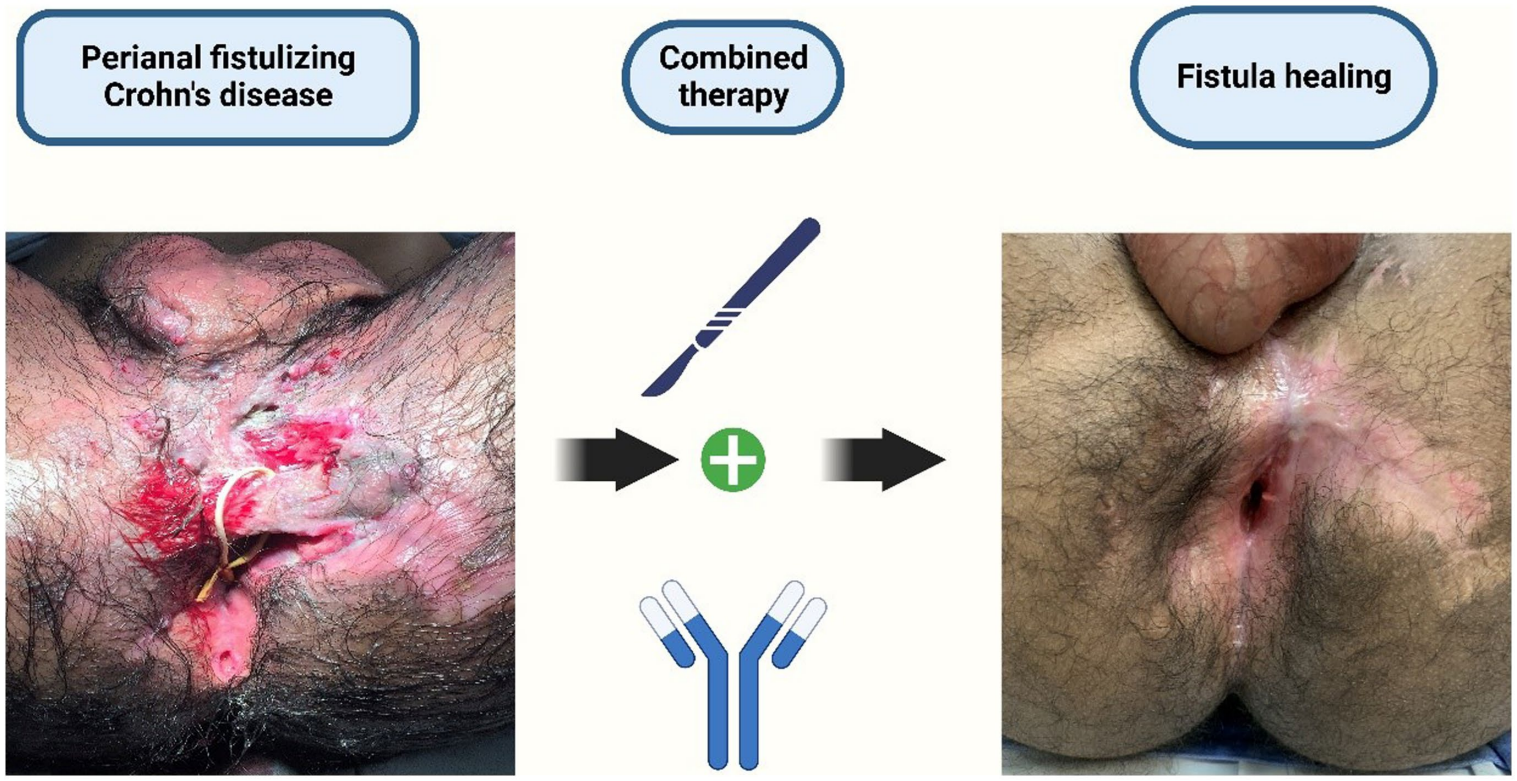
Treatment of perianal fistulizing CD: a combined surgical and medical approach. Created with BioRender.com.

The treatment of perianal fistulizing CD represents a challenge and generally demands a combined surgical and medical approach (see Figure 1). The primary objective of the surgery is to treat and prevent perianal sepsis through examination under anesthesia and seton drainage, thereby enabling the safe initiation of anti-TNF therapy (8, 9). Despite medical and surgical intervention, a considerable number of patients experience refractory disease, and up to 20% may require proctectomy with a permanent stoma (10). Therefore, the investigation into factors that may influence the outcome of perianal disease is one of the key areas of research in CD (11).

The prevalence of nutritional deficiencies is high among CD patients, with estimated prevalence rates of approximately 65–75% (12). The main reasons for that are related to reduced appetite, self-imposed food avoidance/restriction (13), disordered eating behavior (14, 15), persistent mucosal and systemic inflammation, nutrient malabsorption, increased nutrient requirements, medication side effects, and high gastrointestinal losses through chronic diarrhoea, fistulas, and stomas. It is well established that poor nutritional status is a predictive factor for worse clinical outcomes in CD. It is also a risk factor for hospitalization following emergency department attendance and admission due to infection. Additionally, in patients who require surgery, malnutrition is associated with a greater occurrence of postoperative complications (8, 16, 17).

Considering the negative impacts of malnutrition on the prognosis of surgical CD patients, current guidelines recommend conducting routine preoperative nutritional assessments in all patients requiring surgical intervention. Furthermore, optimizing nutritional status before surgeries is advised whenever feasible (8, 16).

Despite these recommendations, which seem to be more widely followed in abdominal surgeries involving bowel resection, there is a lack of emphasis on clinical practice regarding nutritional assessment and correction of nutritional deficiencies in patients with perianal Crohn’s disease (pCD). This discrepancy could be explained by the fact that most patients undergo minor procedures such as abscess drainage, fistulotomy, and seton placement, which are associated with low rates of postoperative complications. Therefore, the actual impact of nutritional status on fistula healing and the prognosis of patients with pCD still need to be explored.

This review aims to give an overview of nutritional status assessment in pCD patients and review the evidence regarding the influence of nutritional status on the outcomes of pCD patients. We searched for relevant publications using the Medline/PubMed database up to 1 July 2023. The following Medical Subject Heading [MeSH] terms alone or matched with the Boolean operators ‘AND’ or ‘OR’ were used: ‘Crohn’s Disease,’ ‘perianal disease,’ ‘fistulizing disease,’ ‘nutritional status,’ and ‘malnutrition.’

## 2. How to assess nutritional status in pCD patients?

The current evidence regarding nutritional status and pCD is extrapolated mainly from data regarding bowel disease, primarily due to the need for studies explicitly addressing that subset of the disease. However, other factors contribute to limitations in pCD research, such as the heterogeneity of endpoints and the diversity of methods used to evaluate therapy response and fistula healing and activity (18).

According to the *European Society for Clinical Nutrition and Metabolism* (ESPEN), malnutrition can be defined as “a state resulting from lack of intake or uptake of nutrition that leads to altered body composition (decreased fat-free mass) and body cell mass leading to diminished physical and mental function and impaired clinical outcome from disease” (19).

Despite this definition, there is controversy regarding the correct way to assess nutritional status in IBD. Commonly, some authors have used assessment tools and parameters such as body mass index (BMI), unintentional weight loss, and serum albumin levels to define malnutrition (20, 21). Additionally, it is common to consider anemia to indicate nutritional deficiency in IBD.

Zhu et al. (22) analyzed clinical data from 52 patients with pCD and identified low body weight in 44.2% of patients, low albumin in 26.9% of patients, and anemia in 42.3% of patients. Subsequently, the same research group published data from an analysis of 139 patients with perianal fistulizing CD, demonstrating that low BMI (<18.5 Kg/m2) was present in 44.6% of patients and 26.6% of patients presented a history of weight loss. Additionally, low levels of hemoglobin (<12.0 g/dL for men and < 11.0 g/dL for women) and albumin (<3.5 g/dL) were found in 38.8 and 23.7% of patients, respectively (23).

Retrospective analysis of 6,082 CD patients who underwent IBD-related major abdominal surgery in the American College of Surgeons’ National Surgical Quality Improvement Program [ACS-NSQIP] between 2005 and 2012 identified that severe hypoalbuminemia was independently associated with a higher risk of 30-day postoperative complications. Low BMI and obesity were also associated with worse postoperative outcomes. Perianal surgeries were not included, and the percentage of patients with perianal disease was also not described (24).

Hypoalbuminemia was also independently associated with 90-day postoperative intra-abdominal septic complication in a retrospective analysis of 815 CD patients who underwent ileocolic resection at Cleveland Clinic Foundation. In total, 33.9% of the patients had prior perianal disease and 17.1% had perianal disease at the time of surgery (25).

Although body weight, BMI, albumin, and hemoglobin levels are commonly used to assess nutritional status, they may not always provide accurate interpretations. Malnutrition can also be expressed with overweight, obesity, and sarcopenia (26). Individuals with IBD experience disturbed body composition, depending on the severity and duration of the illness (27). They tend to exhibit a reduction in lean mass and an increase in obesity over time, rendering body weight and BMI unreliable indicators. Assessing BMI in isolation may overlook essential factors such as body composition, muscle strength, and serum micronutrients, which are often altered in CD patients (21).

Nardone et al. (28) retrospectively evaluated the presence of sarcopenia in a cohort of 63 CD patients with moderate-to-severe clinical activity who underwent CT enterography. In total, 23.8% of patients presented perianal lesions in this cohort. They detected sarcopenia signs in 68.3% of patients. Sarcopenia was associated with a higher risk of infections within 1 year (28). Furthermore, myopenia has also been associated with primary non-response to anti-TNF therapy (29).

Albumin levels tend to be altered in inflammatory conditions as serum albumin decreases due to increased capillary permeability, clearance, and liver synthesis alterations (24, 30). Moreover, its relatively long half-life of approximately 20 days may not accurately reflect the current nutritional status, rendering it unreliable for evaluating responses to nutritional interventions (20).

Anemia is considered the most common extraintestinal manifestation in IBD patients, and it is primarily linked to iron deficiency, chronic disease, and mixed origins. Iron deficiency typically stems from intestinal blood loss due to ulcerated mucosa, reduced intake of dietary iron sources, and impaired mucosal absorption. On the other hand, the anemia of chronic disease is associated with chronic inflammation, leading to disruptions in iron metabolism, reduced erythropoietin production, and inhibition of erythropoiesis (31).

Frequently, there is an overlap between iron deficiency and anemia of chronic disease. Consequently, isolated measurements of hemoglobin levels and blood counts are unreliable for diagnosing iron deficiency anemia. The diagnosis of iron deficiency requires considering the level of inflammatory activity and should include an evaluation of complete blood count, ferritin levels, transferrin saturation, and C-reactive protein (17).

The ESPEN guidelines recommend that IBD patients should be screened for malnutrition at diagnosis and regularly thereafter. It is emphasized that this assessment should be performed with validated tools, such as the *Nutritional Risk Screening-2002* (NRS-2002) and the *Malnutrition Universal Screening Tool* (MUST) (19, 32). However, these assessment tools were not specifically designed and tailored for IBD patients. Table 1 presents some examples of nutritional screening tools used in patients with IBD.

**Table 1:** Nutrition screening tools for patients with IBD.

Not specifically developed for IBD	Specifically developed for IBD
NRS-2002	MIRT
MUST	SaskIBD-NR
GLIM	NS-IBD
	IBD-NST

Some pieces of evidence have demonstrated that NRS-2002 is associated with a missing rate of patients at potential malnutrition risk. Wang et al. (33) retrospectively analyzed clinical data from 146 CD patients. They showed that the malnutrition prevalence rate was 59.6 and 82.2% when NRS-2002 and MUST were used as the first step of the *Global Leader Initiative on Malnutrition* (GLIM) criteria assessment (33).

Recently, the Global Leadership Initiative on Malnutrition (GLIM) criteria have been validated in patients with IBD (34). The GLIM criteria analyze aetiologic and phenotypic parameters in patients at risk of malnutrition and grade malnutrition according to phenotypic criteria (35). Li et al. (36) conducted a retrospective nutritional assessment of 108 CD patients using NRS-2002 and GLIM criteria. The prevalence of perianal lesions in that population was 54.6%. According to NRS-2002 criteria, high nutrition risk was present in 46.3% of participants. Approximately 70% of patients were classified as malnourished when applying the GLIM criteria. The presence of perianal disease was not associated with a higher risk of malnutrition (36).

Developing a nutritional screening tool tailored explicitly for Crohn’s disease, which considers the intricacies of the chronic inflammatory state, is a natural path in research. Jansen et al. (37) proposed a screening tool called “Malnutrition Inflammation Risk Toll (MIRT)” based on BMI, unintentional weight loss over 3 months, and C-reactive protein. They evaluated 55 CD patients in remission, and the screening tool successfully predicted clinical outcomes such as flares, hospitalizations, and surgeries (37). Nevertheless, the tool requires validation in larger and more diverse populations, including those with active disease settings.

Another example of a nutritional screening tool specifically developed for IBD is the “*Saskatchewan Inflammatory Bowel Disease–Nutrition Risk Tool*” (SaskIBD-NR). The SaskIBD-NR assesses four components as follows: gastrointestinal symptoms (nausea, vomiting, and diarrhoea), unintentional weight loss, anorexia, and food restrictions. In the initial study, 110 patients with IBD were screened using SaskIBD-NR, and it demonstrated greater sensitivity, specificity, positive predictive value, and negative predictive value than MUST. Among the evaluated population, 14.7% were found to have perianal disease (38).

In parallel, Camilla Fiorindi et al. (39) have proposed a new screening tool for patients with IBD requiring surgery—the IBD Nutritional Screening tool (NS-IBD). This tool is based on BMI, weight loss, gastrointestinal symptoms, ileostomy presence, and previous surgery history. They found the NS-IBD tool to have increased sensitivity in screening malnutrition, compared with SASKIBD-NR, MUST, Malnutrition Screening Tool (MST), NRS-2002, and MIRT (39).

Wall et al. (40) recently proposed a patient-centered tool, validated for digital use in the outpatient setting that enables nutrition self-screening. The study involved 282 patients with IBD, of whom 175 had CD, but the percentage of perianal disease was not described. The “IBD-specific nutrition self-screening” tool (IBD-NST) evaluated the following components: BMI, weight loss, IBD symptoms, and nutritional concerns. Among the analyzed population, the tool identified 30% of patients at moderate or high nutrition risk (40). One of the possible advantages of this new tool is the ability to identify patients at nutrition risk who are likely to benefit from specialized dietetic assessment and intervention.

Another crucial point that needs to be addressed in an eventual screening tool specific for Crohn’s disease (CD) is the impact of disease location and phenotype on nutritional status. The nutritional consequences of jejunoileal strictures are expected to differ from those of ileosigmoid fistula, pancolitis, or a complex fistulizing perianal disease.

Finally, rather than considering nutritional deficiencies purely as a consequence of IBD, it is time to recognize the nutritional status and dietary factors as one of the causal triggers leading to disturbances in the composition of the gut microbiome, altered homeostasis, and chronic inflammation. This creates a vicious cycle between malnutrition and inflammation that unfortunately is still not completely understood (26).

The “Western” dietary pattern, characterized by high consumption of snacks, prepared meals, condiments, and sauces and coupled with a low intake of vegetables and fruits, has been linked to the development of CD (41). Dietary intake and composition also play a pivotal role in influencing and being influenced by the disease activity. Patients with active disease tend to adhere to the Mediterranean diet less than those in remission (42). Conversely, diets with increased intake of fruits and vegetables, reduction of processed meats and refined carbohydrates, and preference for water for hydration are associated with reduced symptoms of IBD (43).

## 3. Influence of nutritional status on perianal disease course

Despite some data indicating a high prevalence of nutritional deficiencies and malnutrition among individuals with pCD, evidence is scarce regarding their repercussions on pCD disease course, fistula healing, and other clinical outcomes (see Table 2).

**Table 2:** Nutritional risk factors and their repercussion in perianal Crohn’s disease.

Nutritional risk factor	Outcome of perianal Crohn’s disease
Low BMI	Poor fistula healing (44) Persistent perianal disease (45)
Obesity	Higher risk to develop perianal disease, earlier and more frequent episodes of fistulas and abscesses (46) Postoperative complications (47)
Visceral adipose tissue	Anorectal fistula activity (48)

Azzam et al. (44) evaluated the clinical data from 61 patients with perianal fistulizing CD treated with anti-TNF agents. By considering the radiological assessment of the fistula response using MRI, they identified that low BMI was the only predictor of poor fistula healing based on the multivariable analyses (44). The therapeutic effect of biologics appears to be influenced by the nutritional status. Therefore, assessing the nutritional status of all patients with CD is crucial before introducing biologics. Additionally, nutritional optimization should be provided to those at nutritional risk (49).

Low BMI is also associated with chronic/recurrent perianal disease in children newly diagnosed with CD. An analysis of data obtained from the Pediatric IBD Collaborative Group Registry Database, a prospective and multicenter American observational registry, revealed that out of 276 patients with perianal disease, 15% developed perianal lesions within 30 days of diagnosis. The study demonstrated that patients with chronic fistulizing disease had significantly lower BMI values at onset (value of *p* of 0.028) and after 2 years (value of *p* of 0.036) than those with resolving disease (45).

Obesity and the role of visceral adipose tissue in intestinal inflammation are also concerning issues in CD. Adipose tissue is an endocrine and immune organ that influences inflammatory processes. Cytokines and adipokines produced in the mesenteric adipose tissue likely play a crucial role in IBD pathogenesis (50). Obesity also seems to be linked with the disease course of IBD and its response to medical therapy (51). Additionally, visceral obesity is associated with a 2.9 times higher risk of surgery, and obese patients are 2.5 times more likely to experience poor surgical outcomes than non-obese patients (52, 53). Obesity also affects the quality of life, as it is independently associated with higher anxiety, depression, fatigue, pain, and worse social function (54).

Regarding pCD, obese patients appear to have a higher likelihood of developing anoperineal disease. Anoperineal abscesses and fistulas tend to be more frequent and occur earlier in obese patients (46). A recent study examined the relationship between perianal fistula activity and abdominal adipose tissue in CD. The study used pelvic MRI and abdominal CT to analyze adipose tissue characteristics and fistula activity scores in 136 patients with perianal fistulizing CD. Patients in the high-activity group showed a higher density of visceral adipose tissue and a higher ratio between visceral adipose tissue and total adipose tissue, suggesting a higher overall inflammatory load (48).

Regarding postoperative outcomes in obese patients, Manne et al. (47) conducted a case–control study that demonstrated a trend toward poorer surgical outcomes (postoperative wound infection, delayed wound healing or prolonged recovery, wound dehiscence, and development of an abscess or death) among obese patients. However, this analysis did not reach statistical significance (47).

The available evidence concerning the impact of nutritional status on the outcomes of patients with pCD is still limited and primarily derived from studies focused on luminal disease. Even a dedicated and universally validated instrument for evaluating the nutritional status of pCD patients is currently lacking. Investigations regarding the healing of fistulas and therapy response in pCD should henceforth incorporate nutritional status as a pertinent variable. Establishing the correlation between nutritional disorders and the prognosis of pCD patients would open a door to implementing nutritional therapy as an additional strategy to manage the perianal disease, thereby enhancing the overall quality of life for these individuals. Moreover, substantial scope exists for exploration in areas such as the role of dietary factors, body composition, and microbiota in triggering and perpetuating inflammation.

## 4. Conclusion

Although data is limited, the incidence of nutritional disorders seems comparably significant in perianal disease as in luminal disease. Moreover, these disorders appear to correlate with the earlier onset and more aggressive progression of perianal disease. There is a need for nutritional assessment tools specifically tailored for Crohn’s disease patients, taking into account several factors such as body composition, quality of dietary pattern, disease activity, disease phenotype, and location. Further studies are required to determine the true impact of nutritional status on the pathogenesis and course of perianal Crohn’s disease.

## Author contributions

BA: Conceptualization, Methodology, Resources, Writing – original draft, Writing – review & editing. OF: Conceptualization, Writing – original draft, Writing – review & editing. AL: Writing – review & editing. RF: Writing – review & editing. CS: Writing – review & editing. GL: Writing – review & editing. DS: Writing – review & editing. JS: Conceptualization, Supervision, Writing – original draft, Writing – review & editing.

## Funding

The author(s) declare that no financial support was received for the research, authorship, and/or publication of this article.

## Conflict of interest

The authors declare that the research was conducted in the absence of any commercial or financial relationships that could be construed as a potential conflict of interest.

## Publisher’s note

All claims expressed in this article are solely those of the authors and do not necessarily represent those of their affiliated organizations, or those of the publisher, the editors and the reviewers. Any product that may be evaluated in this article, or claim that may be made by its manufacturer, is not guaranteed or endorsed by the publisher.

## Abbreviations

BMI: body mass index
CD: Crohn’s disease
ESPEN: European Society for Clinical Nutrition and Metabolism.
GLIM: Global Leader Initiative on Malnutrition
IBD: inflammatory bowel disease
IBD-NST: IBD-specific nutrition self-screening tool.
MST: malnutrition screening tool.
MUST: malnutrition universal screening tool.
NRS-2002: nutritional risk screening
NS-IBD: IBD nutritional screening tool
pCD: perianal Crohn’s disease
SaskIBD-NR: Saskatchewan Inflammatory Bowel Disease–Nutrition Risk Tool
